# High Incidence of Guillain-Barré Syndrome in Children, Bangladesh

**DOI:** 10.3201/eid1707.101999

**Published:** 2011-07

**Authors:** Zhahirul Islam, Bart C. Jacobs, Mohammad B. Islam, Quazi D. Mohammad, Sergei Diorditsa, Hubert P. Endtz

**Affiliations:** Author affiliations: International Centre for Diarrheal Disease Research, Dhaka, Bangladesh (Z. Islam, M.B. Islam, H.P. Endtz);; University Medical Centre, Rotterdam, the Netherlands (Z. Islam, B.C. Jacobs, H.P. Endtz);; Dhaka Medical College and Hospital, Dhaka (Q.D. Mohammad);; World Health Organization, Dhaka (S. Diorditsa)

**Keywords:** Guillain-Barré syndrome, Campylobacter jejuni, acute flaccid paralysis, infection, children, bacteria, Bangladesh, letter

**To the Editor**: Bangladesh has achieved remarkable success in its drive to eliminate poliomyelitis; no case has been reported from that country since 2000. Still, the nonpolio incidence rate of acute flaccid paralysis (AFP) in Bangladesh is 3.25 cases per 100,000 children <15 years of age (*1*). Guillain–Barré syndrome (GBS), an acute polyradiculoneuropathy, is the most frequent cause of AFP (*2*). GBS in Bangladesh is frequently preceded by an enteric infection caused by *Campylobacter jejuni* (*3*). Frequent exposure to enteric pathogens at an early age may increase the incidence of GBS. We hypothesized that most AFP cases in Bangladesh can be diagnosed as GBS. Our objective was to estimate the crude incidence rate of GBS among children <15 years of age in Bangladesh.

In collaboration with the World Health Organization (WHO), the Government of Bangladesh conducts active AFP surveillance. AFP is defined as acute onset of focal or general flaccid (hypotonic) weakness without other obvious cause (e.g., trauma) in children <15 years of age. Data on the number of reported AFP cases in Bangladesh during 2006 and 2007 were obtained. On the basis of clinical and other information routinely collected through the surveillance system, we defined a GBS case as presence of an acute flaccid (hypotonic) paralysis and symmetrical weakness (*4*) in the absence of injury or birth trauma.

Bangladesh is divided into 6 divisions (major administrative regions) comprising 64 districts. Crude incidence data for GBS were calculated per division and per district on the basis of the population <15 years of age reported by WHO and the Government of Bangladesh.

In 2006 and 2007, a total of 1,619 and 1,844 AFP cases, respectively, were reported in children <15 years of age, of which 608 (37%) and 855 (46%) cases, respectively, fulfilled the GBS case definition. The crude incidence rate for GBS in children <15 years of age varied from 1.5 to 1.7 cases per 100,000 population in the 3 northern divisions (Dhaka, Rajshahi, and Sylhet) and from 2.1 to 2.5 per 100,000 in the 3 southern divisions (Khulna, Barisal, and Chittagong) (Figure A1). Overall, the crude incidence rate of GBS in children <15 years of age varied from 1.5 to 2.5 cases per 100,000 population per year in the 6 divisions of Bangladesh. Incidence rates were high (>5.0/100,000) in the Meherpur and Barisal districts in southern Bangladesh. We found a seasonal fluctuation in the frequency of patients with GBS; the most cases occurred in May (n = 159) and the lowest in February (n = 84). GBS occurred predominantly among boys (59%).

Most incidence studies reported in the literature originate from Europe and North America (*5*). A recent review reported that the best estimate of the global incidence of GBS in children <15 years of age is 0.6 cases per 100,000 population per year (*5*). Reports on incidence rates in developing countries are scarce. The crude incidence rate of GBS in Bangladesh in children <15 years of age reported here appears to be 2.5× to 4× higher than that reported in the literature. A statistically significant proportion of AFP cases in Bangladesh appear to be caused by GBS. We cannot explain the difference in incidence rates of GBS in the 3 northern divisions and the 3 coastal divisions in southern Bangladesh, in which rates are higher. However, differences in incidence rates between northern and southern Bangladesh may be explained by difference in climate or epidemiology. Further research is required to determine why GBS would occur more frequently in southern Bangladesh. Population-based data correlate well with a smaller scale hospital-based study in Khulna (*6*), in which GBS was diagnosed in 47% of hospitalized AFP case-patients. Our recent hospital-based study shows that 25% of GBS case-patients in Bangladesh are children <15 years of age (*3*). It is important to highlight that the present population-based analysis is based on a simplified case definition for GBS. All 25 children in the hospital study in whom GBS was diagnosed on the basis of National Institute of Neurological Disorders criteria (*7*) were also detected through population-based surveillance and fulfilled the simplified GBS case definition. Future studies, however, should include a validation of the case definition used here.

The possibility that GBS is related to pandemic (H1N1) 2009 and the affiliated vaccination campaign has resurfaced recently (*8*). For successful surveillance of excess GBS cases after pandemic (H1N1) 2009 and for postmarketing surveillance of the safety of new vaccines in general, data on the background incidence of GBS are critical. This report indicates that the effect of GBS in Bangladesh is substantial and suggests that data obtained through the ongoing global AFP surveillance program can be used to obtain crude incidence data on GBS worldwide.

## Figures and Tables

**Figure A1:**
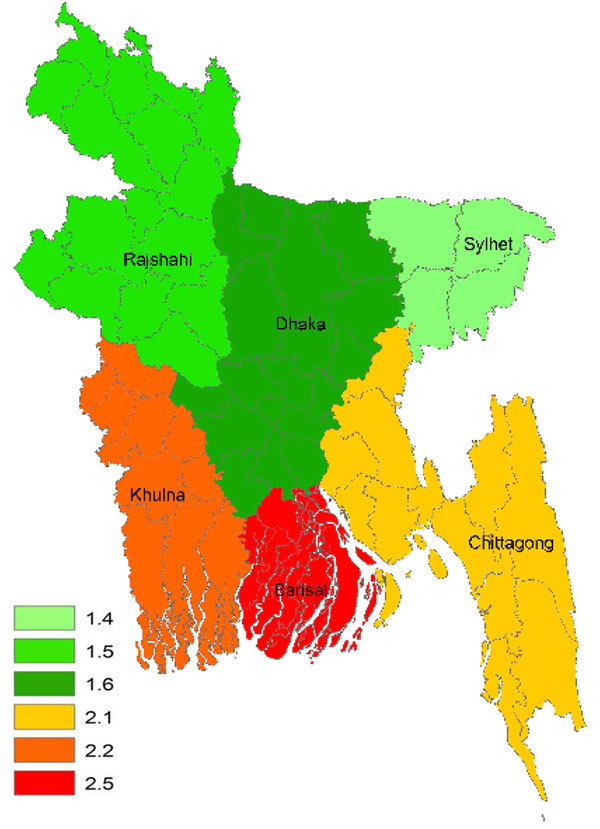
Crude incidence rate (per 100,000 population/year) of Guillain-Barré syndrome in children <15 years of age, Bangladesh, 2006 and 2007.
